# A Panel of VHH Antibodies Against Sabin Type 1 Poliovirus D-Antigen Reveals Strain-Specific and Cross-Serotype Reactivity

**DOI:** 10.3390/v18080833

**Published:** 2026-07-29

**Authors:** Maya Ermakova, Sergey Ivanov, Olga Shmeleva, Nadezhda Borisenko, Anna Zyrina, Stepan Chumakov, Igor Levin, Regina Yakupova, Marina Merkulova, Anna Shishova, Yury Ivin, Anastasia Piniaeva, Alexandra Siniugina, Aydar Ishmukhametov

**Affiliations:** 1 Chumakov Federal Scientific Center for Research and Development of Immune-and-Biological Products of Russian Academy of Sciences (Institute of Poliomyelitis), Moscow 108819, Russia; 2 Shemyakin-Ovchinnikov Institute of Bioorganic Chemistry, Russian Academy of Sciences (IBCh RAS), Moscow 117997, Russia; 3 Institute for Translational Medicine and Biotechnology, First Moscow State Medical University (Sechenov University), Moscow 117418, Russia

**Keywords:** poliovirus, Sabin strains, monoclonal antibodies, VHH, phage display, D- and C/H-antigens, ELISA test, neutralization, polio vaccines

## Abstract

Vaccination remains the primary strategy for poliomyelitis prevention. The D-antigen of poliovirus is a critical component of inactivated polio vaccines, as it induces the production of neutralizing antibodies and provides protective immunity. Therefore, the development of quantitative immunoassays for monitoring D-antigen content during vaccine production is an important task. In this study, we generated recombinant camelid single-domain antibodies (VHHs) specific for the D-antigen of Sabin poliovirus type 1 and evaluated their antigen specificity. The obtained VHH antibodies demonstrated strong binding to the native D-antigen of Sabin type 1 poliovirus. Furthermore, the use of clone S1-C7 as a capture antibody in combination with Sabin type 1-specific polyclonal antibodies for detection revealed differential recognition of the vaccine-derived Sabin type 1 and homologous wild-type Mahoney strains. Notably, such discrimination was not observed when polyclonal antibodies were used alone, indicating that incorporation of VHH S1-C7 into the assay enhances strain-specific antigen recognition. In a neutralization assay, clone S1-C7 exhibited in vitro neutralizing activity against Sabin type 1 poliovirus. One clone, S1-D9, demonstrated cross-reactivity with all three poliovirus serotypes, suggesting recognition of a conserved epitope in the capsid and potential utility as a universal detection antibody. The generated VHH antibodies represent promising analytical tools for poliovirus antigen characterization. Together with S1-C7, they enable the discrimination of both D- and C-antigen forms as well as vaccine-derived and wild-type strains.

## 1. Introduction

Poliomyelitis is a highly contagious disease caused by poliovirus that can result in symptoms ranging from mild flu-like illness to irreversible paralysis and death. Poliovirus belongs to the *Enterovirus coxsackiepol* species of the *Picornaviridae* family [1]. Despite global success in controlling the disease through vaccination, cases of infection associated with wild poliovirus (WPV) and vaccine-derived poliovirus (VDPV) strains, as well as outbreaks of vaccine-associated paralytic poliomyelitis (VAPP), continue to be reported in many regions [2]. Vaccine-associated cases are linked to the use of oral polio vaccine (OPV) containing live-attenuated poliovirus strains, which, in rare cases, can revert to neurovirulent variants [3]. Consequently, the inactivated poliovirus vaccine (IPV) remains an essential component in the global polio eradication strategy [4,5]. It is important to have analytical tools for proper antigen characterization during vaccine manufacture and therefore specific antibodies to create such tools [6].

The critical component of the IPV is the D-antigen—a conformationally native protein capsid of the virion that induces the production of neutralizing antibodies [7]. The poliovirus D-antigen immunogenicity is determined by the structural organization of its icosahedral, symmetrical capsid, which consists of 60 protomeric units. Each protomer includes proteins VP1, VP2, VP3, and VP4, with VP4 located on the interior of the capsid and not involved in the formation of antigenic determinants. VP1, VP2, and VP3 form a network that encompasses the surface of the capsid, with their spatial organization determining the serotype specificity and immunogenicity of the virus [8,9].

A detailed study of poliovirus antigenic structure allowed identification of the four main antigenic sites targeted by virus-neutralizing antibodies. Antigenic Site 1 (AgS1) is immunodominant and is represented by a sequential linear epitope of the major capsid protein, VP1 [10]. Antigenic Site 2 (AgS2) includes amino acid residues of the VP1 and VP2 proteins and is localized in the region surrounding the “canyon”. Antigenic Site 3 (AgS3) is mapped to the VP3 protein, while Antigenic Site 4 (AgS4) is mapped to both the VP2 and VP3 proteins [11]. The differences among neutralizing epitopes have been analyzed by sequencing the genomes of mutants resistant to neutralization with monoclonal antibodies [12,13].

During the poliovirus replication cycle, several types of RNA-free empty capsids are formed. These particles differ in protein composition and immunogenicity. Altered capsid forms generally induce substantially lower levels of neutralizing antibodies. In addition to naturally occurring C-antigen particles, poliovirus particles may undergo conversion to the H-antigen form under stress conditions such as heating (e.g., up to 56 °C) or formalin treatment during IPV production [14,15].

The D-antigen assay is traditionally performed using ELISA, with calibration based on WHO international standards and in-house reagents referenced to NIBSC standards [16]. Quantification of poliovirus D-antigen depends on the antibodies used in the assay. Polyclonal antibodies recognize multiple epitopes and may differ in epitope composition between preparations, requiring revalidation for each new batch [17]. Monoclonal antibodies, in turn, possess high specificity for a single epitope, and well-characterized monoclonal antibodies ensure greater assay reproducibility [18,19]. Various selection methods enable the isolation of monoclonal antibodies that recognize neutralizing epitopes uniquely characteristic of the native D-antigen structure.

The VHH (nanobodies) platform, consisting of single-domain fragments of camelid heavy-chain antibodies, was selected for antibody generation. Compared to full-length IgG, VHHs offer several key advantages, including small size (~20 kDa), high stability, and a broad structural repertoire of antigen-binding loops [20]. Their structural stability and solubility are attributed to evolutionarily conserved hydrophilic residues in the framework region 2 (FR2), which reduce the tendency for aggregation in the absence of a light chain. Binding specificity is determined by three complementarity-determining regions (CDRs), among which CDR3—the longest and most variable—often plays a decisive role [21,22].

To efficiently obtain such antibodies, the phage display technique was employed, enabling high-throughput selection of clones with the desired specificity. This method relies on the expression of antibody variable domains on the surface of bacteriophages, with a direct physical linkage between the packaged gene and its protein product [23].

Previous studies have demonstrated the possibility of generating VHH antibodies against poliovirus type 1. Strauss et al. described five VHH antibodies capable of neutralizing wild-type poliovirus type 1 (Mahoney) and the Sabin type 1 vaccine strain in the low nanomolar range. At higher concentrations, these antibodies completely blocked viral replication [24,25]. In subsequent studies, neutralization-escape mutants were selected for each VHH [26]. High-resolution cryo-EM structural studies revealed that their CDR3 loop penetrates the capsid canyon, thereby blocking receptor binding [27]. However, these antibodies did not discriminate between vaccine and wild-type viruses, limiting their utility for strain-specific analytical applications. In addition, antigenic differences may exist between Sabin type 1 strains by different vaccine manufacturers. This highlights the need for VHH antibodies directed against locally used vaccine seed viruses.

To this end, we selected VHH antibodies that do not interact with other serotypes of vaccine strains or with wild-type viruses. We paid particular attention to the ability of the antibodies to differentiate between D- and C/H-antigens. The obtained VHHs were tested for neutralizing activity and were compared with polyclonal sera in ELISA. In addition, their reactivity toward poliovirus-like particles (VLPs) derived from modified Mahoney capsids was evaluated to assess their applicability for the characterization of alternative poliovirus antigen platforms.

## 2. Materials and Methods

### 2.1. Production and Purification of Poliovirus Type 1 and Alpaca Immunization

The attenuated Sabin poliovirus type 1 (LSc-2ab) strain was used for propagation in Vero cells. The resulting viral suspension was collected and subjected to stepwise concentration by ultrafiltration, achieving a 200-fold increase in virus concentration [28].

The D-antigen of the Sabin poliovirus strain type 1 was purified by gradient ultracentrifugation in cesium chloride (CsCl). For this purpose, the virus containing media was clarified from cellular debris and concentrated by ultracentrifugation through a 30% sucrose cushion (Sigma-Aldrich, St. Louis, MO, USA). The resulting pellet was resuspended in 20 mM PBS (pH 7.2, Chumakov FSC R&D IBP RAS, Moscow, Russia). After removal of residual lipids, the sample was subjected to CsCl (Serva, Heidelberg, Germany; density 1.34 g/cc) gradient ultracentrifugation, and fractions with the highest protein content were collected. Desalting of antigen-containing samples was performed by size-exclusion chromatography on Bio-Gel P-2 columns (Bio-Rad, Hercules, CA, USA) as described previously [29]. The purified virus was inactivated in Medium 199 (FSASI Chumakov FSC R&D IBP RAS, Russia) with 0.03% formaldehyde (PanReac AppliChem, Castellar del Vallès, Spain) at 37 °C for 13 days.

A clinically healthy adult alpaca (*Vicugna pacos*) was maintained throughout the study at a specialized alpaca farm under standard husbandry conditions, with free access to food and water and routine veterinary care. Immunizations and blood collections were performed on site by trained personnel. The animal was monitored after each procedure for potential adverse reactions. The animal was immunized with purified inactivated D-antigen (≥5000 DU, D-antigen units) in Freund’s adjuvant (Pierce, Rockford, IL, USA) by three injections administered at 3-week intervals. Five days after the final immunization, 100 mL of blood was obtained from the jugular vein to isolate lymphocytes. Pre-immune serum was collected on the day of the first immunization, prior to antigen administration.

### 2.2. Construction of a Recombinant VHH Antibody Library

To obtain total RNA and construct a phage library, peripheral blood mononuclear cells (PBMCs) were isolated from the blood of the immunized animal.

The mononuclear cell fraction was purified using a method based on sedimentation in a single-step Ficoll density gradient (Sigma-Aldrich, Darmstadt, Germany). Total RNA was isolated from purified PBMCs using ExtractRNA reagent (Evrogen, Moscow, Russia) according to the manufacturer’s instructions. For cDNA synthesis, reverse transcription was performed using MuMLV reverse transcriptase (Evrogen, Russia). The reaction employed the oligonucleotide primer CH2-IgG–sp_rev (5′-GGTACGTGCTGTTGAACTGTTCC-3′), which is specific to the second constant domain region of alpaca immunoglobulin heavy chains [30]. The resulting cDNA was amplified by PCR using primers containing XbaI and BamHI restriction sites to enable subsequent cloning (Table 1A). The resulting DNA fragments were digested with the restriction endonucleases XbaI (NEB, Ipswich, MA, USA) and BamHI (NEB, USA) and ligated into the pHEN2-XB plasmid vector using T4-ligase (Evrogen, Russia) [31].

Transformation of *E. coli* TG1 (Lucigen, Middleton, WI, USA) cells was performed by electroporation using a Gene Pulser Xcell instrument (Bio-Rad, Hercules, CA, USA). As a transformation control, cells were transformed in parallel with the empty pHEN-XB vector (without the VHH insert) to assess self-ligation background and selection efficiency. During the procedure, a small fraction of transformed cells was serially diluted and plated to determine the number of colonies formed. To determine the library size and transformation efficiency, the number of colonies formed was multiplied by the dilution factor to calculate the number of colony-forming units (CFU). The presence of the target insert in the plasmid was confirmed by PCR analysis of individual colonies (Table 1B).

To obtain recombinant phage particles, *E. coli* TG1 cells containing a plasmid with the target insert were infected with the helper phage M13KO7 (NEB, USA) in Luria–Bertani broth (LB; FSASI Chumakov FSC R&D IBP RAS, Russia) supplemented with 0.1 mM ampicillin and incubated for 4 h at 37 °C. The phages were then precipitated using a PEG-NaCl solution (0.4 M NaCl, 4% PEG-8000). Purified phages were resuspended in 20 mM PBS, mixed with glycerol, and stored at −20 °C. The phage titer was determined by serial dilution of the preparation, followed by infection of *E. coli* TG1 cells and counting of colony-forming units (CFU) on selective medium. The final concentration was calculated using the standard formula:

$$Titer\;(CFU/mL)=\frac{\left(Number\;of\;Colonies\;\times \;Dilution\;Factor\right)}{Volume\;of\;Phage\;Added\;(mL)}$$

Additionally, the presence of VHH inserts in the phagemid was confirmed by PCR analysis of 10 randomly selected colonies using M13-specific primers (Evrogen, Russia). Library diversity was assessed by Sanger sequencing. The nucleotide sequences of 25 randomly selected clones were translated into amino acid sequences and analyzed.

### 2.3. Biopanning of the VHH Library Against the Sabin Type 1 Poliovirus

To select target antibodies, three rounds of biopanning were performed, each consisting of incubation, washing, elution, and re-amplification of bound phages. The D-antigen of each Sabin serotype was adsorbed onto polystyrene tubes at a concentration of 50 DU/mL. To enhance specificity for type 1, a negative selection step was included: the phage library was sequentially pre-incubated with type 2 and 3 antigens to remove cross-reactive variants. After each round, bound phages were eluted with alkaline buffer, used to infect *E. coli* TG1, and plated on YT agar (HiMedia Laboratories, Mumbai, India).

### 2.4. Screening of Recombinant Phages by ELISA and Sequencing of Selected Clones

Following each round of selection, the library was screened for the expression of functional D-antigen-specific antibodies displayed on the surface of M13 phage. Individual colonies of *E. coli* strain TG1 were cultivated in 200 µL of LB medium supplemented with ampicillin (100 µg/mL) to maintain the phagemid harboring the VHH gene fragment for 16–18 h at 37 °C with shaking at 220 rpm. Subsequently, 20 µL of each overnight culture was transferred to deep-well plates containing 800 µL of fresh LB medium and grown at 37 °C with shaking at 220 rpm until an OD600 of ~0.5 was reached (2 h, 37 °C, 220 rpm). Cells were infected with M13/KO7 helper phage (10^12^ PFU/mL) and incubated for 30 min at 37 °C without shaking to allow phage adsorption, followed by 30 min at 37 °C with shaking at 220 rpm. Selection of infected cells was performed by adding kanamycin (100 µg/mL) to the culture, followed by further incubation for 4 h at 37 °C with shaking at 220 rpm. Following incubation, cells were pelleted by centrifugation (4000 rpm, 4 °C, 15 min). The supernatant, containing phage particles, was then transferred to plates that had been sensitized with the D-antigen of three poliovirus serotypes. The antigen was coated at a concentration of 15 µg/mL, as determined by total protein content using the Lowry method. After washing away unbound phages, antigen-bound phage particles were detected using rabbit polyclonal antibodies against the M13 phage p3 protein. Goat anti-rabbit IgG antibodies conjugated to HRP (Thermo Fisher, Waltham, MA, USA) were used as secondary antibodies. Following the addition of TMB substrate, optical density was measured at 450 nm (Multiskan FC, ThermoFisher, USA). Clones were considered positive if their OD_450_ value exceeded the background value of the antigen-free well by more than 40-fold and if they exhibited serotype specificity for the Sabin type 1 poliovirus strain. For clones that passed the binding screen, the VHH-encoding gene region was sequenced using an ABI3500 sequencer (Applied Biosystems, Foster City, CA, USA) by standard Sanger sequencing (BigDye Terminator v3.1, Thermo Fisher Scientific, Vilnius, Lithuania). The resulting sequences were analyzed using SnapGene software (version 1.1.3.) to confirm their uniqueness, correct reading frame, and absence of stop codons and to assess the diversity of the obtained panel of clones.

### 2.5. Expression and Purification of Target Antibodies

Target antibodies were expressed in *E. coli* Rosetta CodonPlus (Novagen, Darmstadt, Germany) harboring a T7-promoter-driven plasmid. Cells were grown in LB medium with ampicillin (100 µg/mL) and chloramphenicol (34 µg/mL) at 37 °C to an OD_600_ of 0.5–1.0, and expression was induced with 1 mM IPTG for 4 h at 37 °C. Cells were harvested, lysed with lysozyme and protease inhibitors, sonicated, and centrifuged.

Purification was performed on an ÄKTA start system (Cytiva, Marlborough, MA, USA) with a HisTrap FF column, 5 mL (Cytiva, USA). After loading, the column was washed with 50 mM Tris-HCl, 300 mM NaCl, and 50 mM imidazole (pH 8.0), and elution was carried out with an imidazole gradient up to 500 mM. Fractions eluting at 200–300 mM imidazole were collected based on A_280_. Purity was assessed by SDS-PAGE and immunoblotting (anti-His-tag), protein concentration was assessed by the Lowry method, and purified antibodies were dialyzed against phosphate buffer using 3.5 kDa Slide-A-Lyzer G3 cassettes (Thermo Fisher, Waltham, MA, USA). In the case of impurity detection, additional purification of antibodies was performed by size-exclusion chromatography on HiPrep 16/60 columns packed with Sephacryl S-100 HR (Cytiva, USA).

### 2.6. ELISA Analysis of the Specificity of Recombinant VHH to Sabin Type 1 Poliovirus

A multistep sandwich ELISA was developed to comprehensively characterize the antigen-binding properties of the obtained VHH antibodies. The selected antibodies were diluted to a concentration of 10 μg/mL in PBS and adsorbed onto the surface of polystyrene 96-well plates (Corning Inc., Corning, CA, USA) at 4 °C for 16 h. At each step, the plates were washed three times with PBS-T (10 mM phosphate buffer, 150 mM NaCl, 0.05% Tween-20, pH 7.4). Blocking was performed using a 1% solution of fetal bovine serum (FBS, Sigma-Aldrich, USA) in PBS-T. To confirm serotype specificity, a panel of purified D-antigens from the three Sabin poliovirus serotypes (LSc 2ab (type 1), P712 Ch 2ab (type 2), and Leon 12a1b (type 3)) and wild-type strains (Mahoney (type 1), MEF-1 (type 2), and Saukett (type 3)) was used. To assess cross-reactivity, a panel of purified D- and H-antigens from the three Sabin serotypes (50 DU/mL) was employed, followed by three-fold serial dilutions in ELISA blocking buffer. Incubation was carried out for 2 h at 37 °C in a thermostatically controlled incubator with humidity control. The detection system consisted of biotinylated rabbit polyclonal antibodies specific to each serotype and horseradish peroxidase (HRP)-conjugated streptavidin (Sigma-Aldrich, USA). Color development was performed using TMB substrate.

### 2.7. Determination of Infectious Virus Titer and Evaluation of the Neutralizing Activity of Recombinant VHH Against Poliovirus

Recombinant VHH antibodies were tested for their neutralizing activity against three attenuated Sabin poliovirus strains (types 1, 2, and 3) and three wild-type poliovirus strains (Mahoney, MEF-1, and Saukett). Prior to the study, virus titers were determined by the endpoint dilution method on Vero cell cultures with visual assessment of cytopathic effect (CPE) in accordance with WHO recommendations [32]. The cell suspension (3 × 10^4^ cells per well) was seeded in 96-well flat-bottom plates in DMEM (Sigma-Aldrich, USA) containing 5% FBS, 2 mM L-glutamine, and antibiotics (100 U/mL penicillin, 100 μg/mL streptomycin). Incubation was carried out at 37 °C in an atmosphere of 5% CO_2_ until a 90–95% cell monolayer formed (24–36 h). The virus-containing fluid was serially diluted (10^1^–10^8^) in maintenance medium (DMEM supplemented with 2% FBS). After removing the culture medium, 100 μL of the appropriate virus dilution was added to each well. Each dilution was tested in eight replicates. A reference strain of the corresponding poliovirus type was used as a positive control, and cells with only the supporting medium were used as a negative control. Incubation was carried out at 37 °C for 5–7 days with daily microscopic examination. CPE was assessed visually according to the following criteria: complete destruction of the monolayer or pronounced cytopathic changes (>75% of cells) were considered a positive result, whereas preservation of an intact monolayer was considered a negative result. Virus titers were calculated using the Karber method:

Titer (log10 TCID_50_/mL) = 1.5 + (total number of wells with CPE)/8

Back-titration was performed to ensure the right infectious dose in the wells of the titration plate. Viruses, according to their titer, were diluted to a single infectious dose of 100 TCID_50_. Antibodies were tested in serial two-fold dilutions starting at 50 ng/mL. A virus-antibody mixture (1:1) was prepared, incubated at 37 °C for 1 h, and added to wells containing cells. The controls included: cells without virus or antibodies (negative control), cells with virus but without antibodies (CPE control), and cells with antibodies but without virus (toxicity control, allowing exclusion of antibody effects on cell viability). Cells were incubated for 7 days at 37 °C in an atmosphere of 5% CO_2_. Cells were examined daily by microscopy to assess cytopathic effect. On day 7, the results were recorded, and wells showing complete suppression of CPE were considered positive for neutralization. The neutralizing antibody titer was defined as the highest antibody dilution at which no CPE was observed in 50% of the wells.

### 2.8. Synthesis and Purification of Poliovirus Virus-like Particles

The genes encoding the VP1, VP3 and VP0 proteins of the poliovirus Mahoney strain with introduced thermostability mutations as described in [33] were cloned into the pFastBac Dual transfer plasmid (ThermoFisher, USA) as described previously [34]. The VP1 gene was cloned under the control of the p10 promoter, while the VP3 gene and the VP0 gene, fused via an autocleavable 2A peptide (from porcine teschovirus-1), were cloned under the control of the polyhedrin (pH) promoter.

The resulting transfer plasmid was transformed into *E. coli* DH10Bac (ThermoFisher, USA) using the heat shock method. Recombinant bacmid DNA was propagated and isolated using the PureLink™ HiPure Plasmid DNA Purification Kit (Invitrogen, Carlsbad, CA, USA). Recombinant baculovirus was generated by transfecting an adherent culture of Sf9 cells (ATCC-CRL-1711) using Lipofectamine 3000 Transfection Reagent (ThermoFisher, USA). Recombinant baculovirus was isolated, amplified, stored, and titrated in accordance with standard methods for the Bac-to-Bac system (ThermoFisher, USA).

To synthesize virus-like particles, suspension Sf9 cells were infected with the recombinant baculovirus at 0.5 PFU (plaque-forming unit)/cell multiplicity of infection and incubated in LeInsect SF9 Medium (QuaCell, China) at 27 °C. Upon observation of a cytopathic effect (4–5 days), the cells were separated from the culture medium by centrifugation (1000× *g*, 5 min) and cell lysis was performed using I-PER Insect Cell Protein Extraction Reagent (ThermoFisher, USA) in the presence of a protease inhibitor cocktail (Halt™ Protease and Phosphatase Inhibitor Cocktail (100×), ThermoFisher, USA).

Virus-like particles were purified by two-step ultracentrifugation using sucrose solutions. First, the cell lysate was ultracentrifuged through a 3 mL 30% sucrose cushion (in PBS) using an SW 32 Ti rotor in an Optima XPN-100 centrifuge (Beckman Coulter, Brea, CA, USA) at 28,000 rpm and 4 °C for 4 h. The resulting pellet was resuspended in PBS and subjected to ultracentrifugation on a 15–45% (*w*/*v*) linear sucrose gradient using an SW 32 Ti rotor at 32,000 rpm and 4 °C for 16 h. The contents of the ultracentrifuge tubes were collected by puncturing the bottom of the tube, and the obtained fractions were analyzed by Western blot hybridization. The sucrose fraction containing the viral protein signal was pelleted by ultracentrifugation using an SW 40 Ti rotor at 28,000 rpm and 4 °C for 4 h and then resuspended in PBS.

## 3. Results

**Figure 1:**
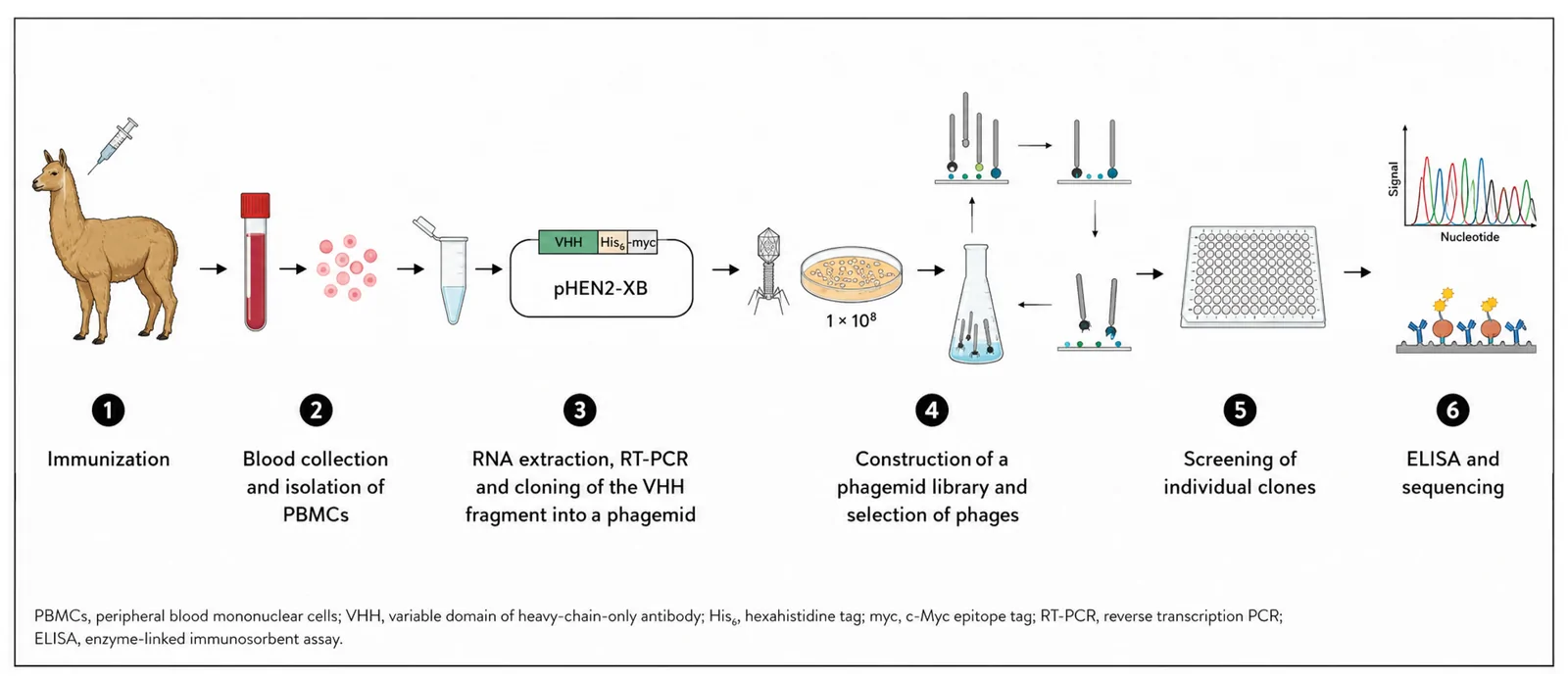
Schematic representation of the construction and selection of a phage library of VHH antibodies against poliovirus Sabin type 1. Created in BioRender. Shishova, A. (2026) https://BioRender.com/hboouqd.

An immune phage library was generated to select recombinant VHH antibodies with high specificity for the Sabin strain poliovirus D-antigen. Figure 1 schematically illustrates the construction and selection of a phage library of VHH antibodies against Sabin type 1 poliovirus.

The process included purification of poliovirus D-antigen and immunization of alpaca to induce an immune response; blood collection and isolation of PBMCs to obtain *B*-lymphocytes; isolation of total RNA and preparation of cDNA for amplification of VHH sequences; cloning of DNA fragments into the phagemid vector pHEN2-XB; transformation into *E. coli* TG1 cells and construction of phagemid libraries; production of phage particles displaying the target antibodies and their selection by biopanning; and screening of individual clones by Sanger sequencing followed by assessment of their specificity by ELISA.

### 3.1. Construction of the Immune Library

Purified D-antigen of the Sabin type 1 poliovirus LSc-2ab strain was used to immunize an alpaca, resulting in the production of polyclonal antibodies. Serum testing revealed the presence of antigen-specific antibodies in the immunized animal, with a titer of 1:2048 after the third immunization round.

### 3.2. VHH Phagemid Library Characterization

**Figure 2:**
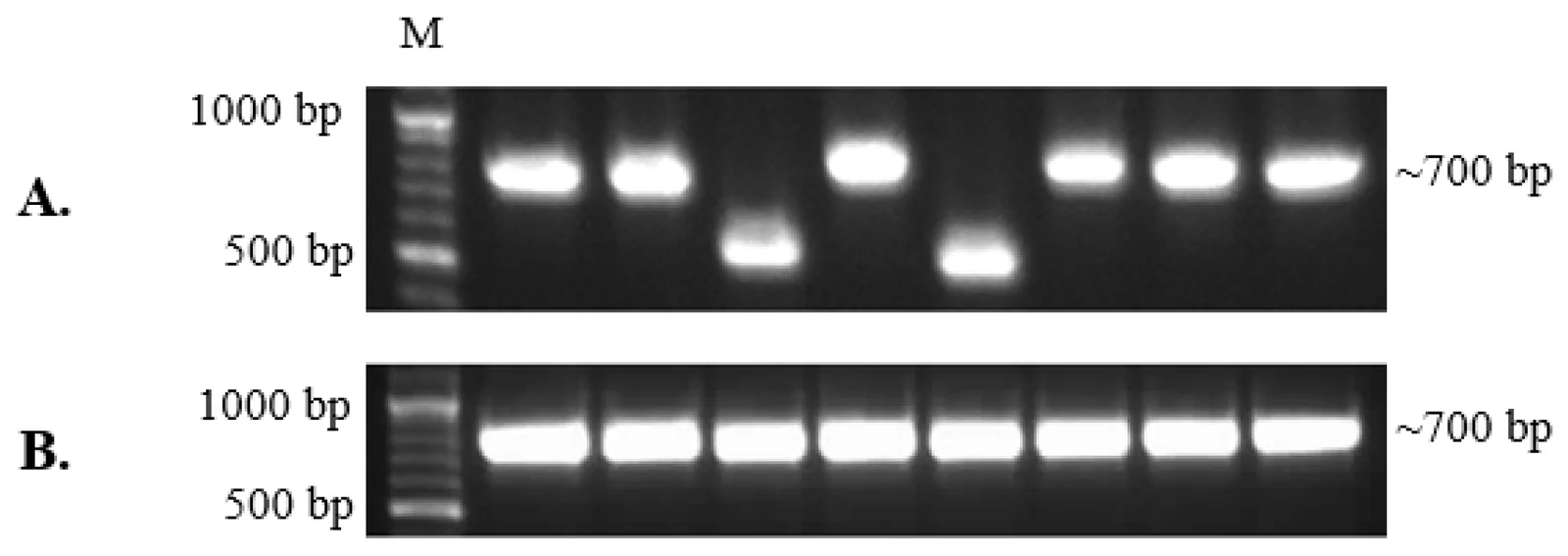
Analytical gel electrophoresis of colony PCR products, M—DNA size marker (1kb, Evrogen, Russia): (**A**) after primary transformation of the phagemid library; (**B**) after the third round of selection.

Transformation yielded an immune VHH library of approximately 7 × 10^7^ CFU/mL. To assess library diversity, 25 randomly selected clones were analyzed by Sanger sequencing, which showed that 23 samples (92%) contained correctly assembled inserts. These sequences contained unique CDR3 regions that determine the specificity, affinity, and functional properties of the antibodies. Colony PCR was additionally performed on 10–20 randomly selected clones after library construction and after each round of biopanning to monitor the presence of full-length VHH inserts and the enrichment of insert-containing clones. Figure 2 shows representative gel images rather than the complete PCR dataset. (Figure 2).

In the representative set shown after primary transformation, 6 of the 8 displayed colonies produced the expected ~700 bp amplicon, whereas two yielded a shorter product of approximately 500 bp (Figure 2A). Following the third round of biopanning, all colonies included in the colony PCR analysis produced the expected ~700 bp amplicon; a representative gel is shown in Figure 2B.

### 3.3. Construction of a Phagemid-Based Phage Library

The phagemid library enables the generation of phage particles displaying the target proteins on their surface as fusions with the p3 coat protein. For the primary round of biopanning, transformed *E. coli* TG1 cells were infected with the helper phage M13/KO7. As a result of the conversion, a phage display library with a titer of 1 × 10^13^ CFU/mL was obtained, suitable for subsequent rounds of affinity selection.

**Figure 3:**
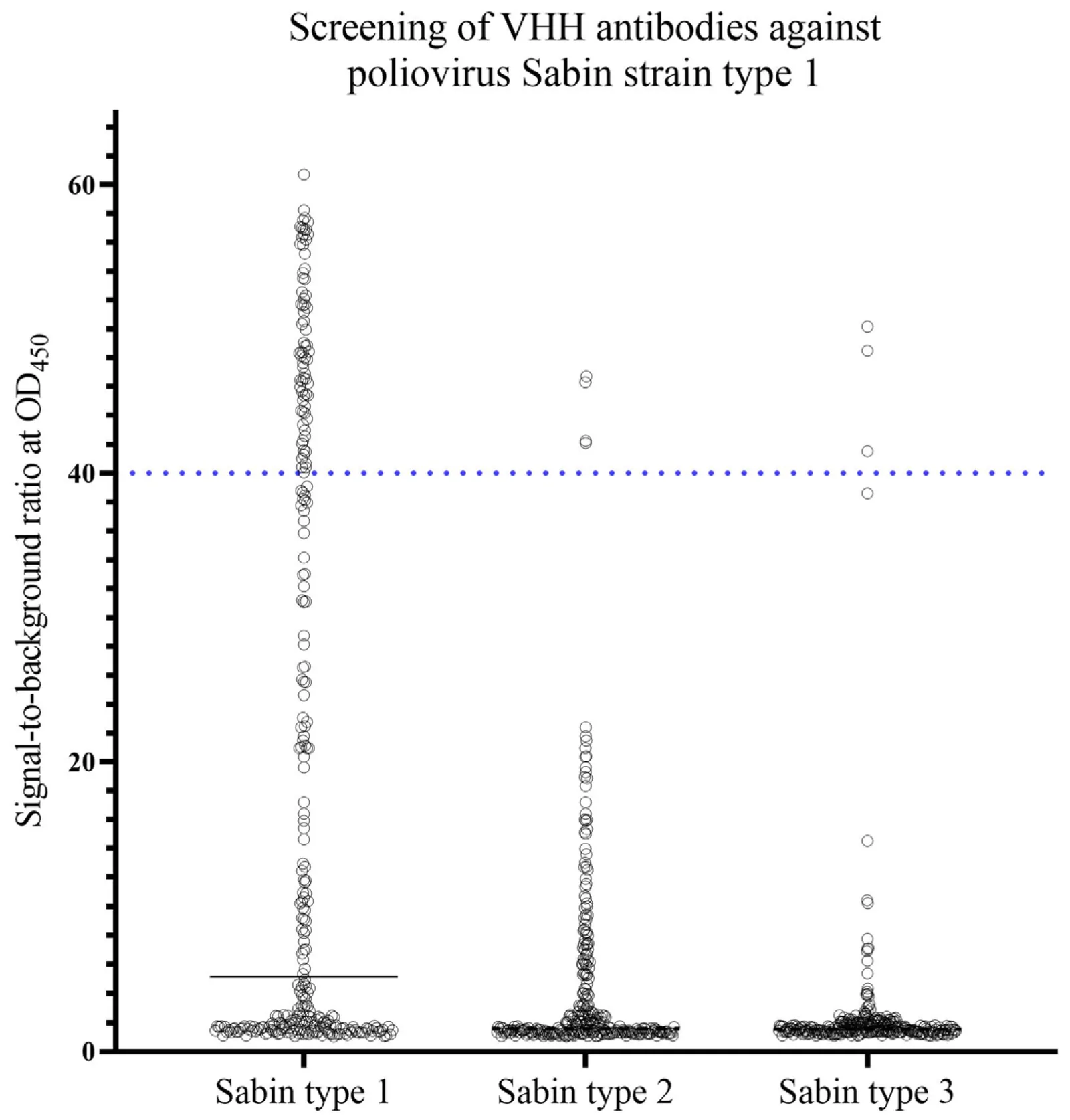
Pools of VHH antibodies selected against poliovirus Sabin strain type 1 during three rounds of biopanning were analyzed for binding to the target antigen—the purified D-antigen of poliovirus Sabin type 1—as well as to antigens of the other two serotypes (types 2 and 3) to assess cross-reactivity. The optical density (OD_450_) of each sample was normalized to the background value (0.055), and the signal-to-background ratio was calculated. Data are shown as individual values for each clone. The selection threshold was set at a signal-to-background ratio ≥ 40 for binding to the target antigen; clones with a ratio above this value and with no cross-reactivity to the other serotypes were selected for further analysis. Each circle corresponds to individual clone signal.

After three rounds of affinity selection, individual clones were screened by ELISA. A total of 288 clones were analyzed, of which 79 (27.4%) showed a significant increase in the signal-to-background ratio (≥40-fold) upon specific binding to the target antigen, inactivated Sabin type 1 poliovirus strain (Figure 3).

Positive clones showed no cross-reactivity with related poliovirus serotypes (types 2 and 3) or with control samples. All 79 specific clones were selected for nucleotide sequencing of the VHH variable domains to identify unique CDR regions. During selection, several clones showed affinity for all three poliovirus serotypes. These were also selected for further analysis.

### 3.4. Amino Acid Sequence Analysis of Selected VHH Clones

**Figure 4:**
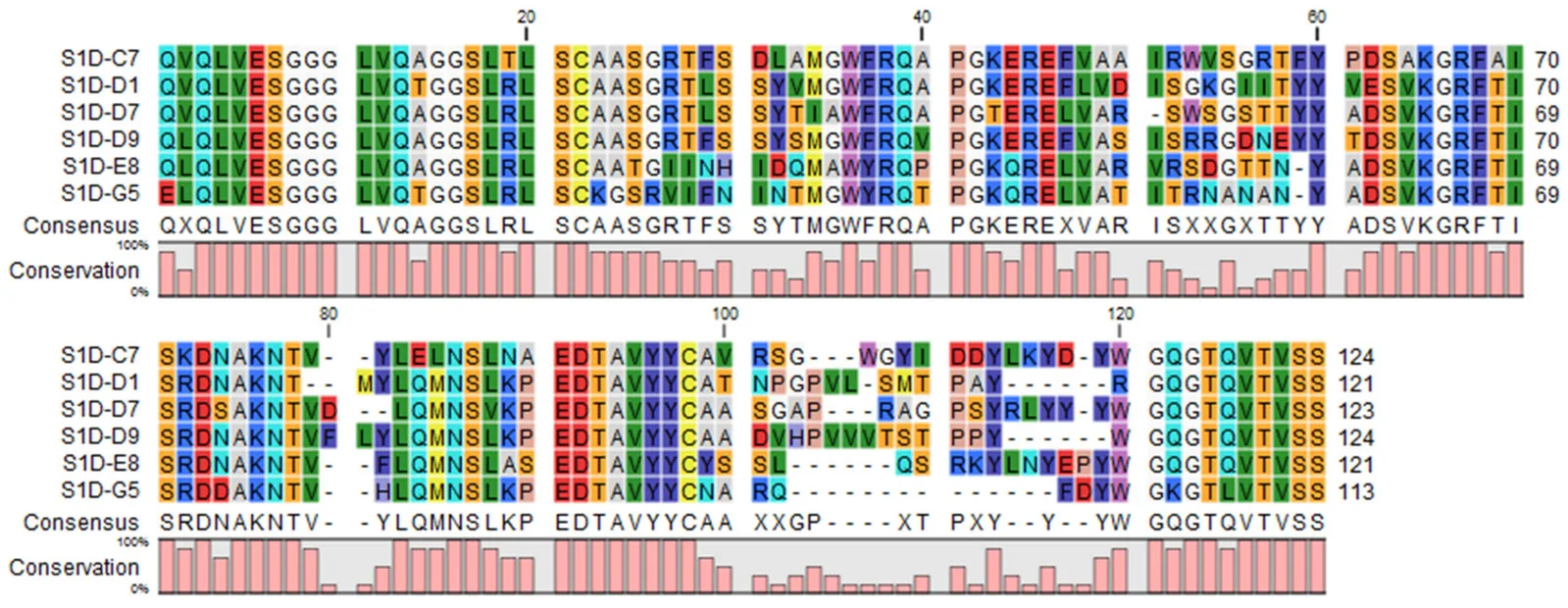
Multiple sequence alignment of recombinant VHH clone amino acid sequences visualized using CLC Sequence Viewer (QIAGEN, Germantown, MD, USA).

Amino acid sequence analysis revealed six unique VHH fragments with significant diversity in length (approximately 120 amino acids) and in the amino acid composition of the CDR3 hypervariable loops (Figure 4).

All obtained sequences contain characteristic structural elements of camelid single-domain antibodies, including four conserved framework regions (FR1–FR4) and three hypervariable loops (CDR1–CDR3). The FR2 region of all clones contains hydrophilic amino acid residues characteristic of VHH antibodies (Gly, Phe, Glu), confirming that they belong to the class of heavy-chain antibodies. These substitutions, evolutionarily conserved in camelids, are critical for maintaining the solubility of VHH fragments in the absence of light chains. CDR3 was the most variable loop, with lengths ranging from 12 to 18 amino acid residues. The diversity of the identified sequences indicates successful enrichment of the library during biopanning and enables the selection of promising candidates for further evaluation of their specificity by ELISA and their neutralizing activity.

### 3.5. Recombinant VHH Antibody Production and Purification

**Figure 5:**
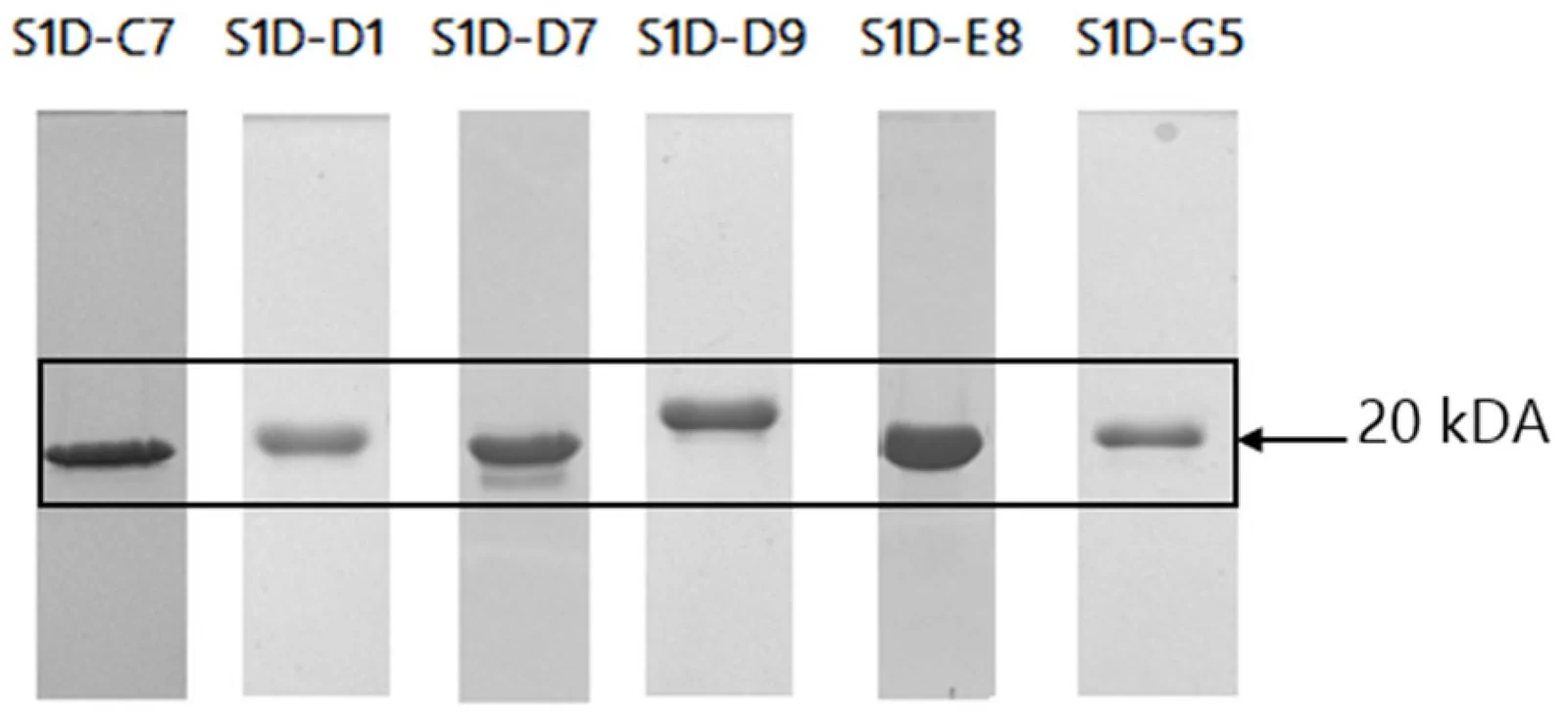
SDS–PAGE analysis of purified VHH antibodies (20% gel) under denaturing conditions. The expected molecular weight of the target protein is ~20 kDa.

To confirm the antigen-binding activity of VHH antibodies in the absence of phage particles, six selected clones were expressed in *E. coli* Rosetta CodonPlus cells following induction with 1 mM IPTG. The antibodies were purified from cell lysates by immobilized metal affinity chromatography (IMAC) using Ni-NTA resin and subsequently subjected to size exclusion chromatography. Electrophoretic analysis confirmed successful purification of monomeric VHH antibodies with an expected molecular weight of approximately 20 kDa (Figure 5).

Sample purity was essential to exclude potential interference from bacterial proteins during ELISA and virus neutralization assays. Representative uncropped SDS-PAGE gels corresponding to the purified antibody preparations are provided in Supplementary Figures S1–S3.

### 3.6. Analysis of the Specificity of VHH Antibodies to Sabin Type 1 Poliovirus in ELISA

**Figure 6:**
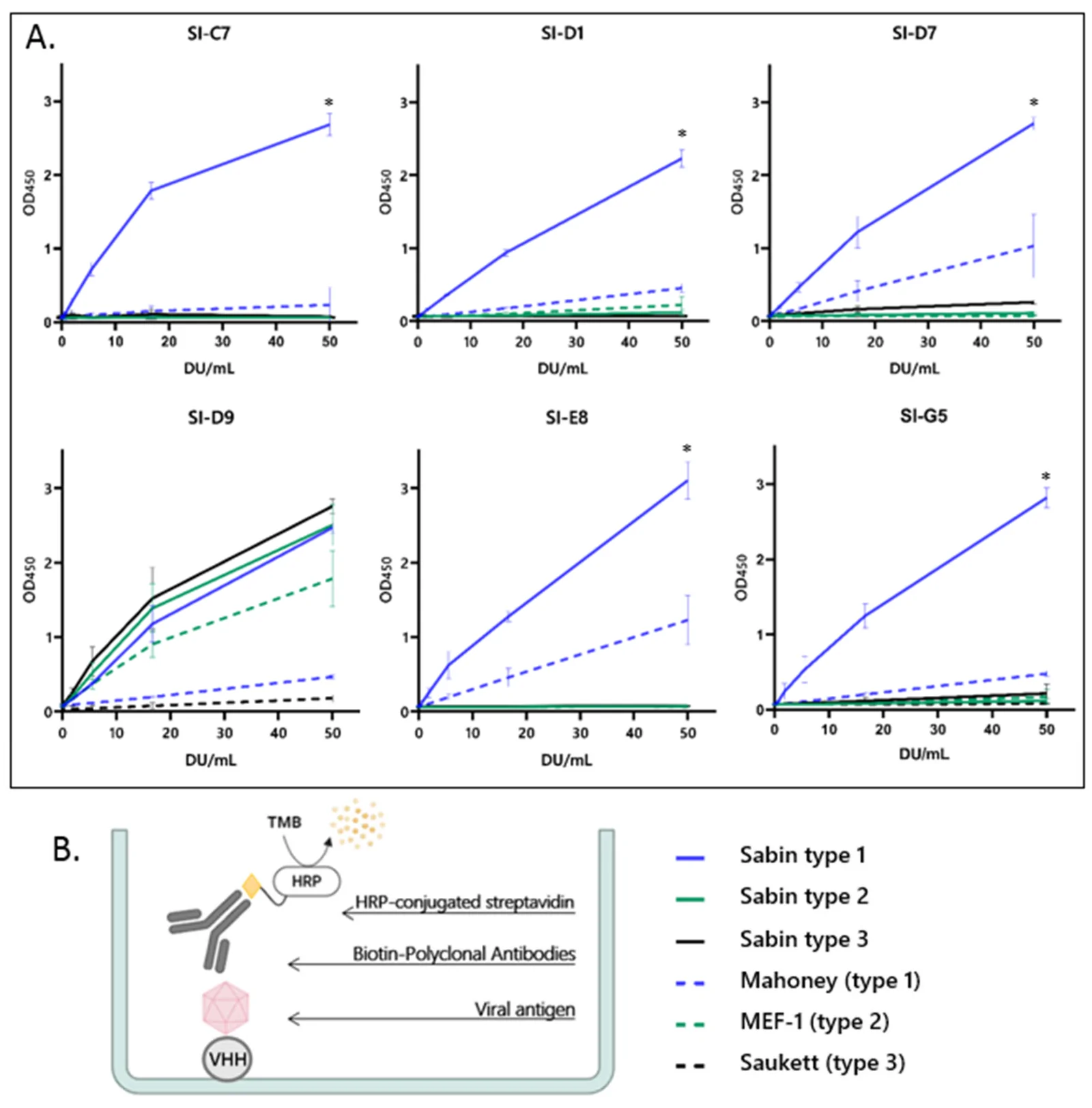
(**A**) Specificity of recombinant VHH antibodies against Sabin type 1 poliovirus as determined by ELISA. The figure shows OD_450_ values as a function of antigen concentration (DU/mL) for monoclonal antibodies S1-C7, S1-D1, S1-D7, S1-D9, S1-E8, and S1-G5. Legend: solid blue line—D-antigen of Sabin type 1; solid green line—D-antigen of Sabin type 2; solid black line—D-antigen of Sabin type 3; dashed blue line—inactivated poliovirus, Mahoney strain; dashed green line—inactivated poliovirus, MEF-1 strain; dashed black line—inactivated poliovirus, Saukett strain. ELISA results were visualized using GraphPad Prism 8.2.1. software,. (**B**) Schematic representation of the ELISA used to determine the specificity of the obtained VHH antibodies against Sabin type 1 poliovirus. Recombinant VHH antibodies were tested for reactivity with D-antigens of Sabin poliovirus strains (types 1, 2, and 3) and wild-type strains (Mahoney, MEF-1, and Saukett), with detection using polyclonal antibodies specific for the corresponding serotypes. Results are presented as means ± standard deviation from three independent experiments (*n* = 3). Asterisks indicate statistical significance calculated using two-way ANOVA. Schematic representation of the ELISA was created using BioRender.com. Shishova, A. (2026) https://BioRender.com/xfsxfpf.

The antigen-binding activity of the obtained VHH antibodies was assessed by ELISA according to the scheme shown in Figure 6B. The purified inactivated D-antigens of three attenuated Sabin poliovirus strains and three wild-type strains (Mahoney, MEF-1, and Saukett) were used as antigens. The initial concentration (DU/mL, D-antigen units) of the D-antigens for all viruses was known, having been determined by ELISA using polyclonal sera and WHO reference standards.

The ELISA results demonstrated strong serotype specificity for five of the six tested monoclonal VHH antibodies when used as capture antibodies (Figure 6A). These findings indicate that five of the six tested monoclonal VHH antibodies recognize epitopes unique to the Sabin type 1 strain. Thus, antibodies S1-C7, S1-D1, S1-D7, S1-E8, and S1-G5 showed the highest reactivity toward Sabin type 1 poliovirus (OD_450_ ≤ 3.3) and moderate binding to the wild-type Mahoney strain (OD_450_ ≤ 0.2–1.3). The observed cross-reactivity of these antibodies with the wild-type virus within the same serotype may be explained by the structural similarity of conserved capsid regions in wild-type and attenuated strains.

Clone S1-D9 demonstrated cross-reactivity with the D-antigen of Sabin strains types 2 and 3 (OD_450_ ≤ 2.6–3.1), as well as with the wild-type strain MEF-1 (OD_450_ ≤ 1.4). This broad cross-reactivity suggests that the S1-D9 antibody recognizes a highly conserved epitope common to different poliovirus serotypes.

### 3.7. Evaluation of the Toxicity and Virus-Neutralizing Activity of VHH Antibodies

After evaluating the specific activity of the antibodies by ELISA, they were analyzed in a neutralization assay with Sabin poliovirus strains types 1, 2, and 3 and with wild-type polioviruses (Mahoney, MEF-1, and Saukett). For each virus, the titer was predetermined, and the working concentration in the neutralization assay was 100 TCID_50_/mL. Due to the risk of cytotoxic effects of the recombinant antibodies on Vero cell cultures, safe working concentrations were individually determined for each clone. Clone S1-C7 showed no cytotoxicity towards the Vero cells throughout the entire range of concentrations tested. Clones S1-D7 and S1-G5 did not cause cell death up to a concentration of 50 µg/mL which was therefore used as the highest concentration in the neutralization assay. For clones S1-E8, S1-D9 and S1-D1, the highest non-toxic concentrations were 25, 12.5, and 6.25 µg/mL, respectively. The bacterial endotoxin test showed no contamination in the antibody samples. Neutralization assays performed in Vero cell culture demonstrated that clone S1-C7 specifically neutralized Sabin type 1 poliovirus, with a minimum neutralizing concentration of 12.5 µg/mL (625 nM). No neutralizing activity was observed for the remaining VHH clones at the concentrations tested.

### 3.8. Differential Detection of Sabin Type 1 and Mahoney Polioviruses by Sandwich ELISA Using the VHH Antibody S1-C7 as a Capture Reagent

**Figure 7:**
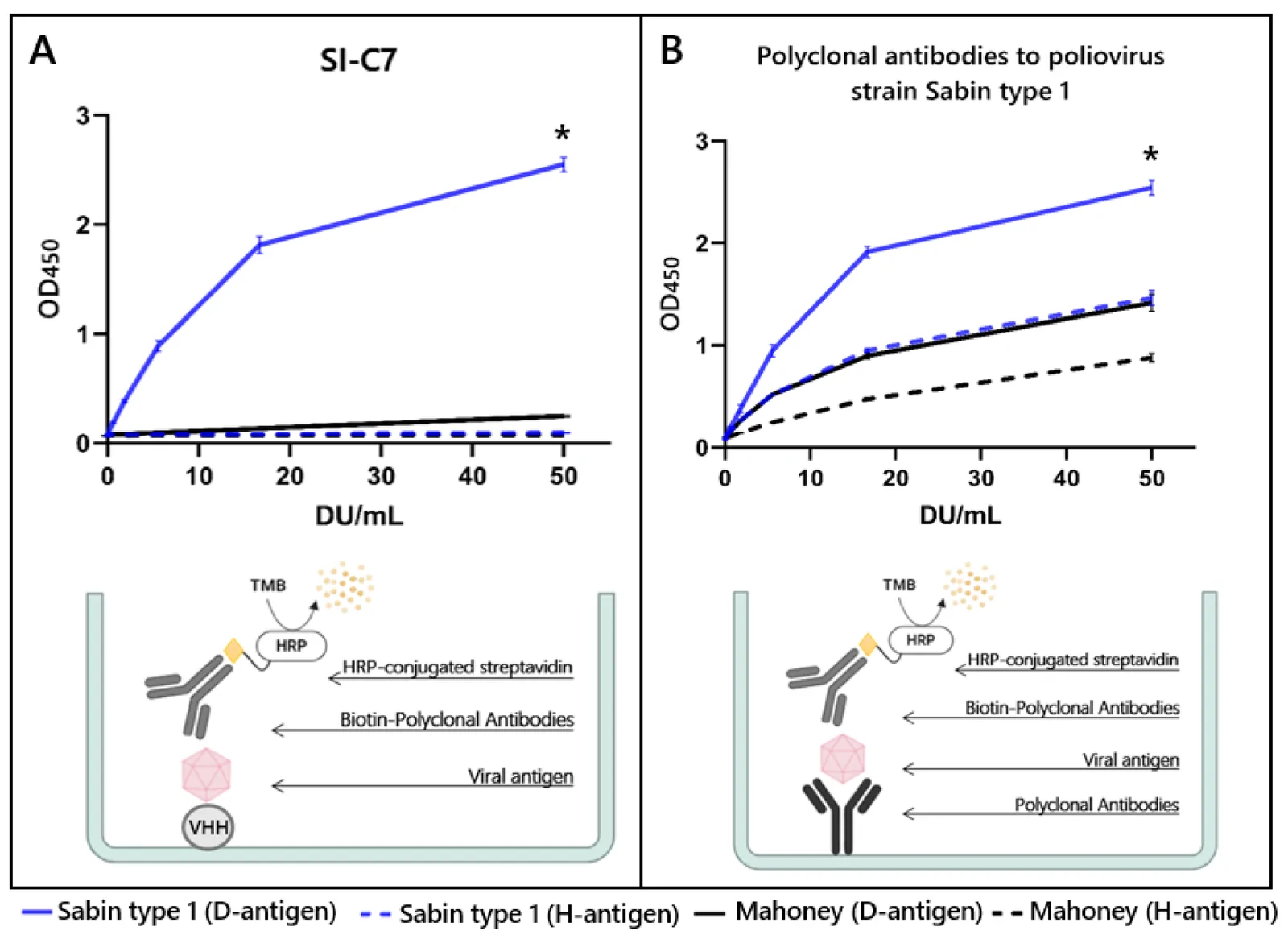
(**A**) Specificity of monoclonal antibody S1-C7 and polyclonal antibodies raised against the Sabin type 1 strain for D- and H-antigens of type 1 polioviruses (Sabin and Mahoney) as determined by ELISA. ELISA scheme with immobilized monoclonal VHH antibodies; (**B**) ELISA scheme with immobilized polyclonal antibodies. Subsequent steps are identical for both panels: addition of antigen (native D-antigen or H-antigen obtained by heat treatment), detection with biotinylated polyclonal antibodies against poliovirus, HRP-conjugated streptavidin, and TMB substrate. The upper graphs show changes in optical density (OD_450_) as a function of poliovirus antigen concentration (DU/mL). The solid line represents the D-antigen, and the dashed line the H-antigen. Blue lines indicate the Sabin strain; black lines indicate the Mahoney strain. (**A**) VHH antibody S1-C7; (**B**) polyclonal serum. Results are presented as means ± standard deviation from three independent experiments (*n* = 3). Asterisks indicate statistical significance calculated using two-way ANOVA. Schematic representation of the ELISA created using BioRender.com. Shishova, A. (2026) https://BioRender.com/9q6mrsb.

The neutralizing antibody S1-C7 was further tested in an experiment alongside polyclonal antibodies to assess its specificity for the Sabin type 1 D-antigen relative to the purified inactivated antigen of the wild-type Mahoney strain, as well as relative to the H-antigens of both the vaccine and wild-type strains. The antigens used included the Sabin type 1 and Mahoney viruses, as well as the same antigens subjected to heat treatment at 56 °C for 1 h to obtain the non-immunogenic H-antigen form. The ELISA schemes and the analysis results are presented in Figure 7. Antibody S1-C7 (Figure 7A) exhibited high specificity exclusively for the D-antigen of the Sabin type 1 poliovirus strain. The polyclonal serum (Figure 7B), containing a mixture of antibodies against various viral epitopes, showed high affinity for the D-antigen as well as binding to the H-antigen (OD_450_ ≤ 1.3) and also interacted with both the D- and H-antigens of the Mahoney virus.

These results demonstrate that the monoclonal VHH S1-C7 when used as a capture antibody is strictly specific for the immunogenic D-antigen of the Sabin type 1 strain, whereas polyclonal antibodies exhibit broader cross-reactivity.

### 3.9. Differential Recognition of Mahoney Virus and Mahoney-Derived VLPs by VHH Antibody S1-C7

**Figure 8:**
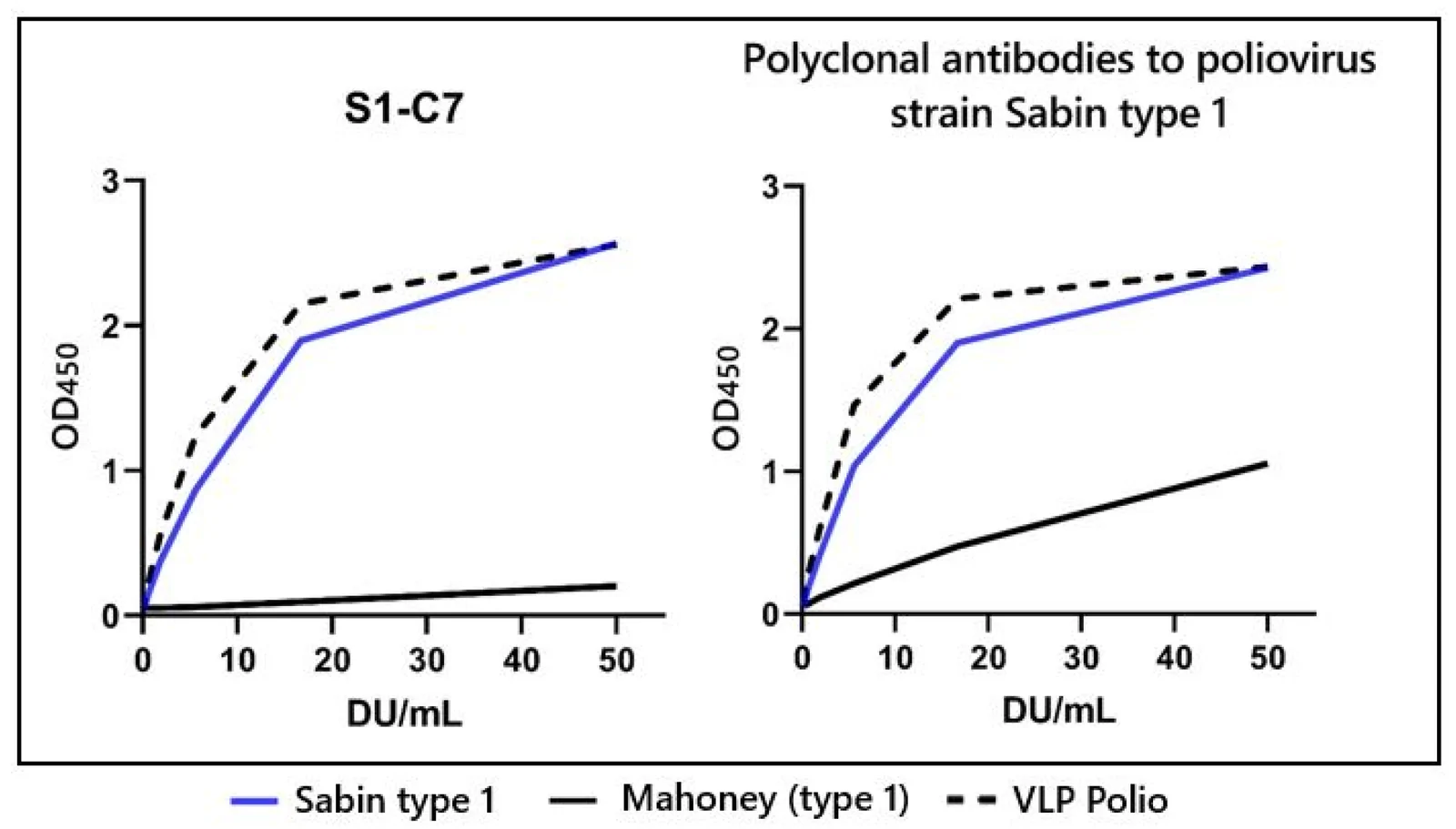
Reactivity of the S1-C7 antibody toward Mahoney-derived virus-like particles (VLPs) in sandwich ELISA. Detection was performed using biotinylated polyclonal antibodies against Sabin type 1 poliovirus. Lines: solid blue—Sabin type 1 D-antigen; solid black—Mahoney D-antigen; dashed black—VLPs.

Antibody S1-C7 was tested as a capture immunosorbent for its reactivity to virus-like particles (VLPs) of the Mahoney-derived poliovirus strain, and the results were compared with those obtained using a polyclonal detection system. Antigens (D-antigen of the Sabin type 1 poliovirus strain, D-antigen of the Mahoney strain (type 1), and Mahoney-derived VLPs) were added starting from an initial concentration of 50 DU/mL and subsequently titrated by serial three-fold dilutions. For detection, biotinylated polyclonal antibodies against the Sabin type 1 poliovirus strain were used in both systems (Figure 8).

In both assays, Mahoney-derived VLPs were efficiently recognized. S1-C7-based sandwich ELISA yielded signals comparable to those obtained with the homologous Sabin type 1 D-antigen. In contrast, only limited reactivity was observed in the S1-C7-based assay with the Mahoney D-antigen, consistent with the strain-specific properties. Notably, the ability of S1-C7 to recognize Mahoney-derived VLPs despite its weak reactivity toward the parental Mahoney virus may reflect structural differences introduced during VLP design or assembly [33].

## 4. Discussion

Monoclonal antibody-based test systems are important analytical tools used for assessing antigen concentration, immunogenicity, and product consistency during vaccine manufacturing.

A previously described approach involved the development of a universal monoclonal antibody-based ELISA test system for the quantitative determination of the D-antigen, intended for use in IPV manufacturing facilities transitioning from wild-type to attenuated Sabin strains [18]. However, the human monoclonal antibodies selected by the authors did not differentiate between wild-type and vaccine poliovirus type 1 strains, and no information was provided regarding the ability of these antibodies to distinguish between D- and C-antigens. In parallel, M. Strauss and colleagues described and characterized VHH antibodies capable of neutralizing both strains (Sabin type 1 and Mahoney). However, those studies were primarily focused on the therapeutic or fundamental investigation of neutralization mechanisms, rather than on analytical specificity for vaccine quality control [25]. Consequently, an important objective of the present study was the development of a highly specific analytical system for the quality control of inactivated polio vaccines based on Sabin strains. For the first time, VHH antibodies (clones S1-C7, S1-D1, and S1-G5) were obtained that specifically recognize the D-antigen of the Sabin type 1 vaccine strain and do not interact with the antigen of the homologous wild-type Mahoney strain. This finding is significant for the accurate quantification of the D-antigen in settings where both Sabin- and Salk-based vaccines are manufactured or controlled.

The high specificity of monoclonal antibodies can be particularly advantageous in sandwich immunoassays when used in combination with polyclonal detection antibodies. Accurate quantification of poliovirus D-antigen requires discrimination from non-native capsid forms, including the C-antigen, as well as from antigenically related wild-type strains. In our study, polyclonal antibodies efficiently detected poliovirus antigens but also showed measurable reactivity with the C-antigen and with the homologous wild-type Mahoney strain (Figure 6 and Figure 7). In contrast, when the monoclonal VHH antibody S1-C7 was used as the capture antibody, the resulting sandwich ELISA demonstrated enhanced discrimination between D- and C-antigens and enabled differentiation of the vaccine-derived Sabin type 1 strain from the wild-type Mahoney strain.

Interestingly, the S1-C7-based sandwich ELISA detected Mahoney-derived VLPs despite showing limited reactivity toward the parental Mahoney strain (Figure 8). Since the VLP construct contains several engineered substitutions in the capsid proteins, these modifications may have altered the antigenic properties of the particles and contributed to their recognition by the antibody system [33]. However, additional studies, including epitope mapping and analysis of engineered mutant particles, will be required to determine whether either substitution directly contributes to S1-C7 binding [35].

The discovery of the cross-specific VHH antibody S1-D9 is of considerable interest for the further development of test systems for poliovirus vaccine quality control (Figure 6). This antibody was found to interact with the D antigens of all three Sabin vaccine serotypes, as well as with the homologous wild type MEF 1 strain (type 2). Therefore, clone S1-D9 represents a promising candidate as a universal detection antibody in a sandwich ELISA, which would allow the replacement of polyclonal sera and simplify assay standardization.

In the virus neutralization assay, one of the six antibodies, S1-C7, specifically neutralized the Sabin type 1 strain. The lack of neutralizing activity of the remaining five clones may be explained by several reasons. First, these antibodies may recognize epitopes that are not involved in virus binding to the CD155 cell receptor or that are located in regions of the capsid that are not critical for viral entry. Second, we cannot rule out that the neutralizing activity of these clones might have been observed at higher concentrations; however, their cytotoxic effects on Vero cell cultures limited concentrations in the media. Our experiments showed that clones S1-E8, S1-D9, and S1-D1 induced cell death at threshold concentrations of 25, 12.5, and 6.25 µg/mL, respectively. This observation raises the important question of the potential toxicity of VHH antibodies, which we plan to investigate in future studies. Notably, the bacterial endotoxin test revealed no contamination in the samples, ruling out classical endotoxin-mediated toxicity.

The recombinant nature of VHH antibodies offers important advantages for vaccine quality control assays. Unlike polyclonal sera, VHHs can be reproduced indefinitely from a defined genetic sequence, ensuring batch-to-batch consistency and eliminating the need for repeated animal immunization [16]. Such properties are particularly valuable for ELISA-based monitoring of poliovirus D-antigen during vaccine manufacture, where assay standardization and long-term reproducibility are critical. In the present study, the VHH antibody S1-C7, when used as a capture antibody in combination with polyclonal detection antibodies, enabled enhanced discrimination between D- and C-antigens as well as between vaccine-derived Sabin type 1 and wild-type Mahoney strains. The selected VHHs displayed diverse CDR3 sequences and distinct antigen-binding profiles, suggesting recognition of different epitopes on the viral capsid (Figure 4). Consequently, combinations of VHH antibodies may provide broader antigen coverage while retaining high analytical specificity, potentially reducing the risk of false-negative results associated with partial antigen denaturation or epitope masking. Furthermore, engineering VHHs into multivalent formats, such as Fc-fused constructs, may increase functional avidity and assay sensitivity. Such approaches could ultimately enable the development of fully recombinant ELISA systems based exclusively on VHH-derived reagents, providing a robust and standardized platform for poliovirus vaccine antigen quantification.

Further validation of the identified VHH antibodies will include direct comparison with established polyclonal and monoclonal reagents used for IPV D-antigen testing. A bridging study could evaluate assay sensitivity, specificity, precision, linearity, and agreement across vaccine lots using the WHO International Standard 17/160 for Sabin IPV D-antigen quantification [16]. Future work will also focus on expanding the antibody panel to other poliovirus serotypes and on generating Fc-fused VHH formats to improve assay flexibility and functional performance.

## 5. Conclusions

Six VHH antibodies against the D-antigen of Sabin type 1 poliovirus were generated and characterized. Clone S1-C7 demonstrated selective recognition of the native D-antigen, enabled discrimination between vaccine-derived and wild-type type 1 polioviruses in sandwich ELISA, and exhibited neutralizing activity. The obtained VHH antibodies represent promising recombinant tools for poliovirus antigen analysis and quality control of inactivated poliovirus vaccines.

## Figures and Tables

**Table 1:** Oligonucleotide primers used in cloning and PCR screening. (**A**) Primers for cloning. (**B**) Primers for PCR screening.

No.	Name	Nucleotide Sequence (5′–3′)
(**A**)		
1	F1X	GATCTCTAGAATGCAGKTGCAGCTCGTGGAGTCNGGNGG
2	R1B	GATCGGATCCGGTTGTGGTTTTGGTGTCTTGGG
3	R2B	GATCGGATCCGGGGGGTCTTCGCTGTGGTGCG
(**B**)		
1	seqM13-F	TGCTTCCGGCTCGTATGTTG
2	seqM13-R	GCATTCCACAGACAGCCCTC

## Data Availability

Raw data is available from the authors upon reasonable request.

## Supplementary Materials

The following supporting information can be downloaded at: https://www.mdpi.com/article/10.3390/v18080833/s1, Figure S1: SDS-PAGE gel showing purification of recombinant VHH clone S1-C7. The band corresponding to the purified VHH antibody (~20 kDa) used for Figure 5 is indicated by the box; Figure S2: SDS-PAGE gel showing purification of recombinant VHH clones S1-E8 and S1-D7. The bands corresponding to the purified VHH antibodies (~20 kDa) used for Figure 5 are indicated by boxes; Figure S3: SDS-PAGE gel showing purification of recombinant VHH clones S1-D1, S1-D9, and S1-G5. The bands corresponding to the purified VHH antibodies (~20 kDa) used for Figure 5 are indicated by the box.
