# Supplementary material for: A Panel of VHH Antibodies Against Sabin Type 1 Poliovirus D-Antigen Reveals Strain-Specific and Cross-Serotype Reactivity
Source: Viruses. 2026 Jul 29;18(8):833. doi: 10.3390/v18080833 (PMC13517935; doi:10.3390/v18080833)
Supplement: Supplementary file 1 [file viruses-18-00833-s001.zip › viruses-4417890-supplementary.pdf]

Article

A Panel of VHH Antibodies against Sabin Type 1 Poliovirus D-Antigen Reveals Strain-Specific and Cross-Serotype Reactivity

Maya Ermakova [1,†*], Sergey Ivanov [1†], Olga Shmeleva [1], Nadezhda Borisenko [1], Anna Zyrina [1], Stepan Chumakov [3], Igor Levin [1], Regina Yakupova [1], Marina Merkulova [1], Anna Shishova [1,2,*], Yury Ivin [1], Anastasia Piniaeva [1], Alexandra Siniugina [1], Aydar Ishmukhametov [1,2]

Supplementary materials

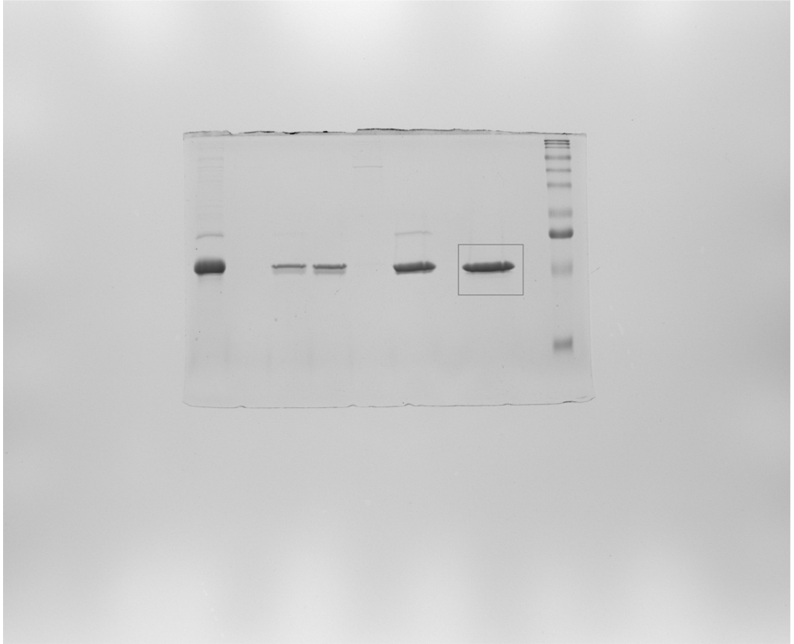

Supplementary Figure S1. SDS-PAGE gel showing purification of recombinant VHH clone S1-C7. The band corresponding to the purified VHH antibody (~20 kDa) used for Figure 5 is indicated by the box.

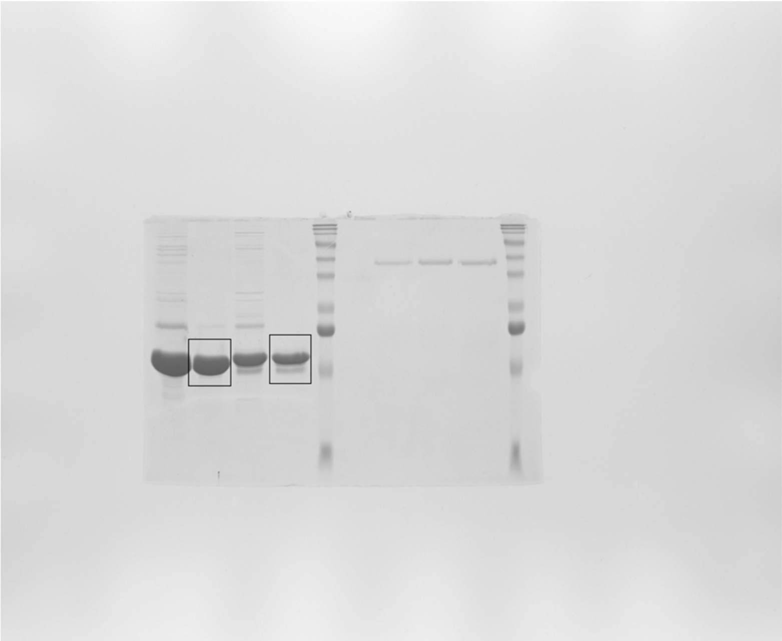

Supplementary Figure S2. SDS-PAGE gel showing purification of recombinant VHH clones S1-E8 and S1-D7. The bands corresponding to the purified VHH antibodies (~20 kDa) used for Figure 5 are indicated by boxes.

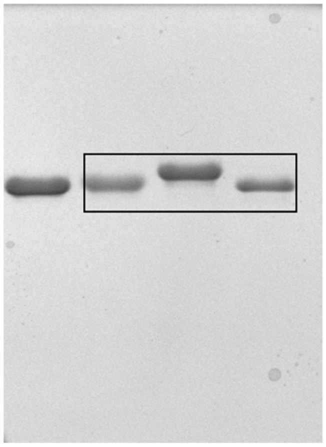

Supplementary Figure S3. SDS-PAGE gel showing purification of recombinant VHH clones S1-D1, S1-D9, and S1-G5. The bands corresponding to the purified VHH antibodies (~20 kDa) used for Figure 5 are indicated by the box.
